# Temporal patterns of energy intake and cognitive function and its decline: a community-based cohort study in China

**DOI:** 10.1093/lifemeta/loac011

**Published:** 2022-07-07

**Authors:** Hui Chen, Yang Tao, Min-Dian Li, Yuxuan Gu, Jiaxi Yang, You Wu, Dongmei Yu, Changzheng Yuan

**Affiliations:** School of Public Health, Zhejiang University School of Medicine, Hangzhou, Zhejiang, China; School of Public Health, Zhejiang University School of Medicine, Hangzhou, Zhejiang, China; Department of Cardiology and the Center for Circadian Metabolism and Cardiovascular Disease, Southwest Hospital, Army Medical University, Chongqing, China; Department of Social Security, Center for Gerontology Research, Nanjing Normal University, Nanjing, Jiangsu, China; Bia-Echo Asia Centre for Reproductive Longevity & Equality (ACRLE), Yong Loo Lin School of Medicine, National University of Singapore, Singapore, Singapore; Department of Obstetrics and Gynaecology, Yong Loo Lin School of Medicine, National University of Singapore, Singapore, Singapore; Institute for Hospital Management, Tsinghua University, Beijing, China; National Institute for Nutrition and Health, Chinese Center for Disease Control and Prevention, Beijing, China; School of Public Health, Zhejiang University School of Medicine, Hangzhou, Zhejiang, China; Department of Nutrition, Harvard T.H. Chan School of Public Health, Boston, MA, United States

**Dear Editor,**

Worldwide, around 55 million people had prevalent dementia in 2019, which is expected to triple by 2050, especially in low- and middle-income countries [1]. Lacking timely diagnosis and limited effective treatment for dementia make identifying risk factors crucial for its early prevention, among which dietary factors have received increasing attention [1].

Recently, accumulating evidence from population-based studies has linked the temporal patterns of energy intake (TPEI), usually defined as the temporal distribution of energy intake during a day, to mortality and metabolic diseases [2], such as diabetes and hypertension. *In vitro* and *in vivo* studies also revealed that meal timing could drive metabolic alterations and circadian regulation [3], and disrupted meal timing altered the peripheral circadian clocks in the hippocampus and consequently affected cognitive function [4]. However, population-level evidence on the association between the TPEI and cognitive function remains lacking. We thus aimed to examine this relationship in the China Health and Nutrition Survey from 1997 to 2006, a community-based cohort study with national representativeness [5].

We included participants aged ≥55 years who completed at least one dietary assessment and cognitive test, and excluded those who: (1) had severe cognitive impairment at baseline (cognitive function score < 7/27); (2) had extreme energy intake (>99th percentile or <1st percentile); (3) had stroke, ischemic attack, hypertension, diabetes, or cancer at baseline. A total of 3342 individuals with up to four repeated measures over 10 years were included for analysis (Supplementary Fig. S1), the mean (standard deviation) baseline age of whom was 62.2 (6.8) years (Table 1), 61.2% lived in rural areas, and 13.6% achieved a high school or higher degree.

**Table 1.** Baseline characteristics of the study participants (*n* = 3342)

Variable^a^	Temporal pattern of energy intake					
	Evenly-distributed	Breakfast-dominant	Lunch-dominant	Snack-rich	Dinner-dominant	Breakfast-skipping
N	1639	364	232	119	278	710
Age (year)	63.0 (7.2)	61.9 (7.1)	61.3 (6.6)	61.2 (7.2)	61.9 (7.2)	61.9 (6.9)
Female (%)	806 (49.2)	181 (49.7)	107 (46.1)	61 (51.3)	133 (47.8)	339 (47.7)
Rural area (%)	983 (60.0)	260 (71.4)	147 (63.4)	63 (52.9)	178 (64.0)	416 (58.6)
Holding a high school degree (%)	193 (11.8)	45 (12.4)	36 (15.5)	41 (34.5)	44 (15.8)	96 (13.5)
Ever smoker (%)	568 (34.7)	130 (35.7)	75 (32.3)	42 (35.3)	96 (34.5)	239 (33.7)
Current alcohol drinker (%)	549 (33.5)	113 (31.0)	86 (37.1)	28 (23.5)	103 (37.1)	261 (36.8)
High physical activity level (%)	433 (26.4)	132 (36.3)	100 (43.1)	64 (53.8)	105 (37.8)	266 (37.5)
BMI ≥24.0 kg/m^2^ (%)	514 (31.4)	134 (36.8)	83 (35.8)	60 (50.4)	79 (28.4)	225 (31.7)
Total energy intake (kcal/d)	2102.9 (561.5)	2021.1 (565.2)	2073.9 (573.1)	2253.3 (521.7)	2080.6 (567.8)	2122.1 (591.0)
Cognitive function (points)	16.2 (4.9)	15.5 (5.1)	16.2 (5.3)	16.6 (4.7)	15.9 (5.0)	15.9 (5.0)

Continuous variables are presented in mean (standard deviation), and categorical variables are presented in *n* (percentage).

Dietary intake was assessed using a combination of weighing methods and a 3-day 24-h dietary recall at each wave. Average daily energy intake from breakfast, morning snack, lunch, afternoon snack, dinner, and evening snack was calculated using the Chinese Food Composition Table [6]. To characterize energy intake distribution across major meals and snacks throughout a day, we identified six TPEIs (Fig. 1a) using the k-means algorithm [7]. Specifically, the “evenly-distributed” pattern was characterized by total energy intake (TEI) approximately evenly spread out among three major meals (28.5%, 36.3%, and 33.8% from breakfast, lunch, and dinner, respectively). Participants with the “breakfast-dominant” pattern had an average of 49.5% TEI from breakfast. Participants with the “lunch-dominant” pattern consumed 64.3% TEI from lunch. Those with the “dinner-dominant” pattern had 64.5% TEI from dinner. The “snack-rich” pattern had 36.8% TEI from snacks. The “breakfast-skipping” pattern was characterized by an average of 5.9% TEI from breakfast. About one-third (33.0%) of participants maintained their patterns from baseline to the end of follow-up. Cognitive function was assessed using the modified Telephone Interview for Cognitive Status (TICS-m), comprising immediate and delayed word recalls (20 points), backward counting (2 points), and serial-7 subtraction test (5 points). The total global cognitive score ranged from 0 to 27, with a higher score representing a better cognitive function.

**Figure 1:**
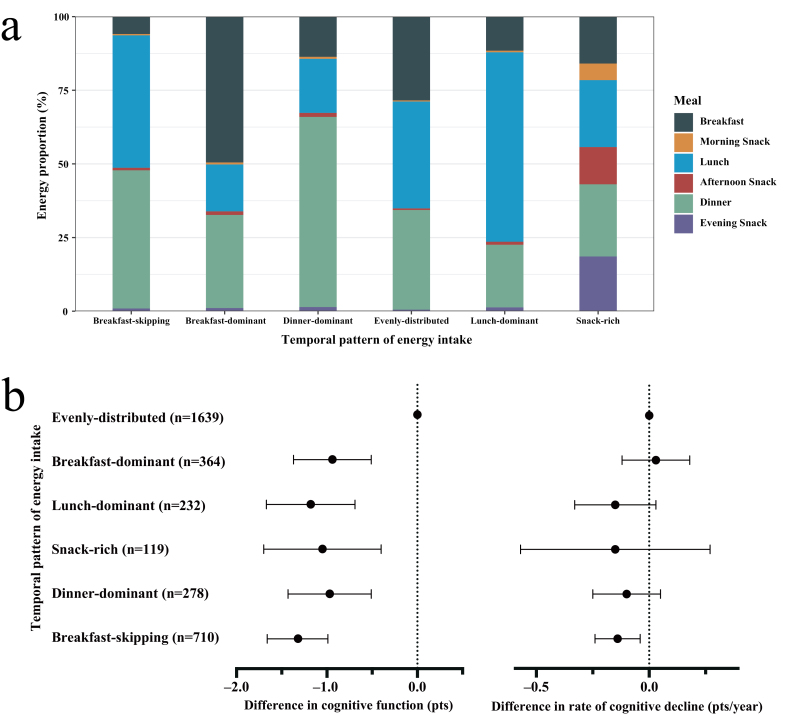
Temporal patterns of energy intake* among participants in the China Health and Nutrition Study (a) and their associations with cognitive function and its decline** (b). *The temporal patterns of energy intake are identified using the k-means algorithm with maximized pseudo-*F*-statistic. **Mixed-effect linear regression was used to estimate the difference in cognitive function and its decline rate associated with different temporal patterns of energy intake, with the evenly-distributed pattern being the reference group. The model was adjusted for age (continuous), sex (female or male), residence (rural or urban), total energy, physical activity (tertiles), smoking status (ever smoker or not), alcohol consumption (currently drinking or not), household income (tertiles), education level (holding high school degree or not), and BMI, (<24, 24.0–27.9, ≥28.0 kg/m^2^).

Next, we assessed the correlation of TPEIs to cognitive function using linear mixed models (LMMs) adjusted for age, sex, residence (rural or urban), total energy, physical activity (tertiles), smoking status (ever smoker or not), alcohol consumption (currently drinking or not), household income (tertiles), education level (below high school or not), and body mass index (BMI) (<24, 24.0–27.9, ≥28.0 kg/m^2^). All other five TPEIs were associated with poorer cognitive function compared with the evenly-distributed pattern (Fig. 1b, Supplementary Table S1), with β (95% confidence interval [CI]) being −0.94 (−1.37, −0.51) for the breakfast-dominant pattern, –1.18 (−1.67, −0.69) for the lunch-dominant pattern, −0.97 (−1.43, −0.51) for the dinner-dominant pattern, −1.05 (−1.70, −0.40) for the snack-rich pattern, and −1.32 (−1.66, −0.99) for the breakfast-skipping pattern. These associations were similar across subgroups defined by sex, age, and residence, but were stronger among individuals with BMI <24 kg/m^2^ and with an education level below high school (Supplementary Table S2). The associations were similar for the verbal memory domain (Supplementary Table S4).

Further, we compared the rate of cognitive decline for participants with different TPEIs using LMMs. Compared with the evenly-distributed pattern, the breakfast-skipping pattern was associated with significantly faster cognitive decline by 0.14 points/year (95% CI: −0.24, −0.04) (Fig. 1b, Supplementary Table S1). No significant association was observed for other patterns. This relation was only significant for individuals aged <65 years (Supplementary Table S3).

These findings were similar in a series of sensitivity analyses. The results persisted when we further adjusted the models for communities in urban or rural areas and overall diet quality. Lagged for one wave, the findings were generally similar, while the relations to cognitive function were nonsignificant for breakfast-dominant pattern and snack-rich pattern, with β (95% CI) being −0.45 (−1.09, 0.20) and −0.63 (−1.53, 0.27), respectively. The lagged association of breakfast-skipping pattern with cognitive decline was also nonsignificant [β (95% CI), −0.18 (−0.41, 0.04)]. When we restricted the analyses to 631 participants who had constant TPEI from baseline to end of follow-up, the association of breakfast-skipping pattern with cognitive function persisted, but its relation to cognitive decline was attenuated to nonsignificant [β (95% CI), −0.09 (−0.27, 0.10)], potentially due to the reduced statistical power. Details can be found in Supplementary Table S4.

When we alternatively classified the TPEIs into evenly-distributed, breakfast-dominant, lunch-dominant, and dinner-dominant using *a priori*-based definition, all other three patterns demonstrated lower cognitive function, compared to the evenly-distributed pattern, whereas none of the patterns showed relations to faster cognitive decline (Supplementary Table S5). We also separately assessed the associations of the energy intake in the morning, noon and afternoon, and evening to cognitive function and its decline (Supplementary Table S6). Higher energy intake in the morning was associated with better cognitive function and a slower decline. The medium quartiles (second to third) of noon and afternoon energy intake also demonstrated protective associations with cognitive function but not with cognitive decline. Evening snack consumers had a significantly slower cognitive decline and better cognitive function (Supplementary Table S7) than nonconsumers.

To our knowledge, this study is one of the few population-based studies that explore the association of TPEI and cognitive decline, although accumulating studies have linked TPEI to health outcomes, including obesity [8], hypertension [9], and cardiovascular health [10]. Emerging studies suggested that meal timing is associated with cognitive function. An experimental study showed that evenly spreading the same amount of energy into four meals can improve short-term cognitive performance than that of two meals [11]. Another meta-analysis [12] including 34 experimental studies showed that breakfast skipping is related to worse acute cognitive function among healthy adults than breakfast consumers. Our findings were generally consistent with prior evidence, showing that breakfast skipping was associated with exceptionally faster cognitive decline than other TPEIs, corroborated by the secondary finding that higher energy intakes in the morning were associated with better cognitive function and slower decline. For snack intake, we observed that only snacks consumed after dinner exhibited a potentially beneficial role, most probably resulting from the fact that people who consumed snacks at night usually used to be brain workers with higher education levels and tended to have a better cognitive function. Our findings should be placed in the context of China’s rapid transitions in eating habits, where accessibility of food choices as snacks may vary significantly across populations.

Several biological mechanisms could explain the observed associations. Meal timing is essential in synchronizing the circadian clock system, particularly for peripheral tissues such as the liver and adipose tissue [13]. Also, meal timing influences critical factors related to cognitive decline, e.g. lipid profile, glucose regulation, insulin resistance, and blood pressure. Furthermore, circadian clocks in the neural circuits related to cognitive functions respond to meal timing. Energy unbalanced eating occasions may result in misalignment between peripheral clocks and the central oscillator, probably through the hypothalamic-pituitary-adrenal axis [14]. Furthermore, skipped breakfast might reflect the evening chronotype, which is related to poorer cognitive performance due to circadian disruption, modulated by corticospinal excitability and cortical facilitation/inhabitation [15]. Besides, energy intake predominantly from one meal might cause abrupt excessive nutrient intake, leading to increased oxidative stress, disrupted synaptic activities, and consequently cognitive impairment.

The strengths of the current study included the prospective design, a well-maintained cohort, reliable dietary assessments, and the temporal sequence of exposure and outcome that reduced potential reserve causation. However, several limitations should be noted. First, the generalizability may be limited since most participants were rural residents with an education level below high school. Second, the dietary data were collected from 1997 to 2006, and whether our findings could be applied to future aging generations warrants further investigation. Moreover, the 3-day 24-hour dietary recall may not sufficiently represent the long-term temporal patterns, and the subjectiveness of mealtime cannot be eliminated since no exact clock time was recorded. Furthermore, the relatively small sample sizes in several patterns might have limited the statistical power to detect significant associations.

In conclusion, we observed that maintaining balanced energy intake across three major meals was associated with significantly better cognitive function than the other five unevenly distributed patterns. In particular, breakfast skipping was associated with significantly worse cognitive function and faster cognitive decline over time. The observed associations were similar across major prespecified subgroups. Further studies are needed to confirm our findings in different populations and reveal the underlying mechanisms. If proven causal, these findings will add to the evidence for future public health recommendations on balanced TPEI for primary prevention of cognitive decline in the aging population.

## Supplementary Material

loac011_suppl_Supplementary_Material
