# Supplementary material for: Temporal patterns of energy intake and cognitive function and its decline: a community-based cohort study in China
Source: Life Metab. 2022 Jul 7;1(1):94–7. doi: 10.1093/lifemeta/loac011 (PMC11749483; doi:10.1093/lifemeta/loac011)
Supplement: loac011_suppl_Supplementary_Material [file loac011_suppl_Supplementary_Material.pdf]

Supplementary Methods

Study design and participants

The China Health and Nutrition Survey (CHNS) is an ongoing open cohort commenced in 1989 with follow-ups every two to four years. Utilizing a multistage random cluster sampling process, participants were selected from sampled counties in nine provinces in mainland China with distinct geographical formations and economic development. Household-based surveys including sociodemographic, lifestyle, and diet were conducted through face-to-face interviews. In addition, cognitive status was measured in four waves from 1997 to 2006 among residents who were 55 years or older. The CHNS was approved by the institutional review committees of the University of North Carolina at Chapel Hill, and the National Institute of Nutrition and Food Safety, Chinese Center for Disease Control and Prevention. Written informed consent was obtained from all participants.

In the current study, we included 4566 participants aged ≥55 years old who had valid baseline dietary data and baseline cognitive function assessments and excluded those who: (1) had severe cognitive impairment at baseline, (cognitive function score <7/27)[1] (n=619); (2) had extreme energy intake (> 99th percentile or < 1st percentile)[2] (n = 36); (3) had stroke, ischemic attack, hypertension, diabetes, or cancer at baseline (n = 569). Eventually, a total of 3342 individuals aged 55-93 years were included for analysis (**Figure S1**). From 1997 to 2006, three waves of follow-up were conducted, with dietary intake and cognitive function being reassessed (**Figure S2)**.

Temporal pattern of energy intake

Dietary intake was assessed using a combination of weighing methods and 3-day 24-hour dietary recall for three consecutive days at each wave. Trained field interviewers recorded food name, amount, type of meal, and place of consumption of all food items consumed in preceding 24 hours every evening for three days. All condiments and cooking oil were also measured using scales by interviewers at the beginning and end of the 3-day survey periods. Energy and nutrient intake was calculated based on the Chinese Food Composition Table[3]. The energy consumption assessment has been validated by the doubly labeled water method with a correlation efficient of 0.56 for men and 0.60 for women[4]. The average amount of energy intake from breakfast, morning snack, lunch, afternoon snack, dinner, and evening snack per day was computed.

TPEI in this study was defined as the pattern of energy intake distribution across major meals and snacks throughout a day. To capture the energy distribution characteristics, we identified six TPEIs using k-means algorithm[5], with the maximization of the pseudo-F statistic being used as the criteria for choosing the optimal cluster number.

Assessment of cognitive function

Cognitive function was assessed by the cognitive score summed from the cognitive screening items used in CHNS. It comprises a subset of items from the modified Telephone Interview for Cognitive Status (TICS-m)[6] and has been used in other Chinese prospective cohort studies[7, 8]. The test was conducted during the face-to-face interview. The cognition assessments included immediate and delayed recalls of a 10-word list (20 points), counting backward from 20 to 1 (2 points), and consecutive subtractions of 7 from 100 (5 points). The total global cognitive score ranged from 0 to 27, with a high cognitive score representing a better cognitive function.

Covariates

Covariates were also obtained from each wave of CHNS interviews, including age, gender, residence, smoking status, alcohol consumption, household income, education level, physical activities, BMI, and communities in urban or rural areas. Physical activities were assessed using metabolic equivalents (MET-hours/week) to standardized activities from work, commuting, and leisure time, weighting each activity by its intensity level and duration. BMI was calculated with weight in kilograms divided by squared height in meters. The proportion of missing data on education level, physical activity, and BMI were 3.8%, 6.1%, and 1.5%, respectively. Mean/mode imputations were used for the missing data in continuous and categorical variables, respectively.

Statistical analysis

We describe the baseline characteristics of participants by TPEI. Mean (standard deviation) was used for normally distributed variables, medium (25th percentile, 75th percentile) was used for variables with non-normal distributions, and percentage (%) was used for categorical variables. One-way analysis of variance and Pearson's χ^2 test was used to test group differences for continuous and categorical variables, respectively.

Longitudinal association of the TPEIs with cognitive function was assessed by mixed-effect linear regression model with the following form:

$$CF_{\{i,t\}} = \beta_0 + u_i + \beta_t t + \beta_P P + \sum_{j=1}^{p} \beta_{X_j} X_j + \varepsilon$$

where i denotes the i-th participants, t denotes the time since baseline assessment, and β_0 is the intercept for population average; u_i denotes the individual-specific random intercept; P is the dummy of TPEI; X_j is the j-th covariates included for confounder adjustments. β_P is the parameter of interest representing the differences of cognitive function of participants with different TPEIs.

Differences in the rate of cognitive decline associated with different TPEIs were tested by additionally including a TPEI by time interaction term $(P \times t)$ in the linear mixed models.

$$CF_{\{i,t\}} = \beta_0 + u_i + \beta_t t + \beta_P P + \beta_{Pt}(P \times t) + \sum_{j=1}^{p} \beta_{X_j} X_j + \varepsilon$$

The coefficient of the cross product term, β_{Pt} is the parameter of interest since it represented the differences of cognitive function change by time (i.e., cognitive decline) in participants with different TPEIs.

We gradually adjusted the models for multiple potential confounding variables: (1) Model 1 was the crude model with no adjustment; (2) Model 2 was adjusted for age and gender (age-squared was not included due to lack of statistical significance); (3) Model 3 was additionally adjusted for residence (rural or urban), total energy, physical activity (tertiles), smoking status (ever smoked or not), alcohol consumption (currently drinking or not), household income (tertiles), education level (below high school or not), and BMI (<24, 24.0-27.9, ≥28.0 kg/m^2). We conducted several subgroup analyses and tested modification by gender, age (<65 years or ≥65 years), BMI (<24 or ≥28.0 kg/m^2), residence (urban or rural area), and education level (below high school or not). Moreover, we assessed the relation of TPEI with the cognitive subdomain of verbal memory.

To test the robustness of our results, we performed several sensitivity analyses. Firstly, communities in urban or rural areas were further included in our model as a covariate to assess the cluster effect on the whole model. Secondly, we estimated the association of TPEI with cognitive

function lagged for one wave (time interval = 2-4 years). Thirdly, we additionally adjusted the Chinese Food Pagoda Score (CFPS) as a diet quality measurement in the model.[9] Lastly, we tested the association among those participants who had repeated dietary measurements and consistent TPEI.

Furthermore, we alternatively categorized energy intake patterns according to a priori-based definition method[10] and classified the study participants into four groups ("evenly-distributed ", "breakfast-dominant", "lunch-dominant", "dinner-dominant"). To explore the individual association of energy intake from morning (breakfast with morning snack), noon and afternoon (lunch with afternoon snack), and evening (dinner with evening snack), with cognitive function and its decline, we included the quartiles of energy intake from the three eating occasions as exposures in the models. Moreover, we explored the association between cognitive function score and energy intake from morning snacks, afternoon snacks, and evening snacks, respectively.

Statistical analyses were conducted using R 4.1.0, with "nlme" package[11] being used to fit the mixed-effect linear models. Tests with two-sided p-values <0.05 were considered statistically significant.

Supplementary Tables and Figures

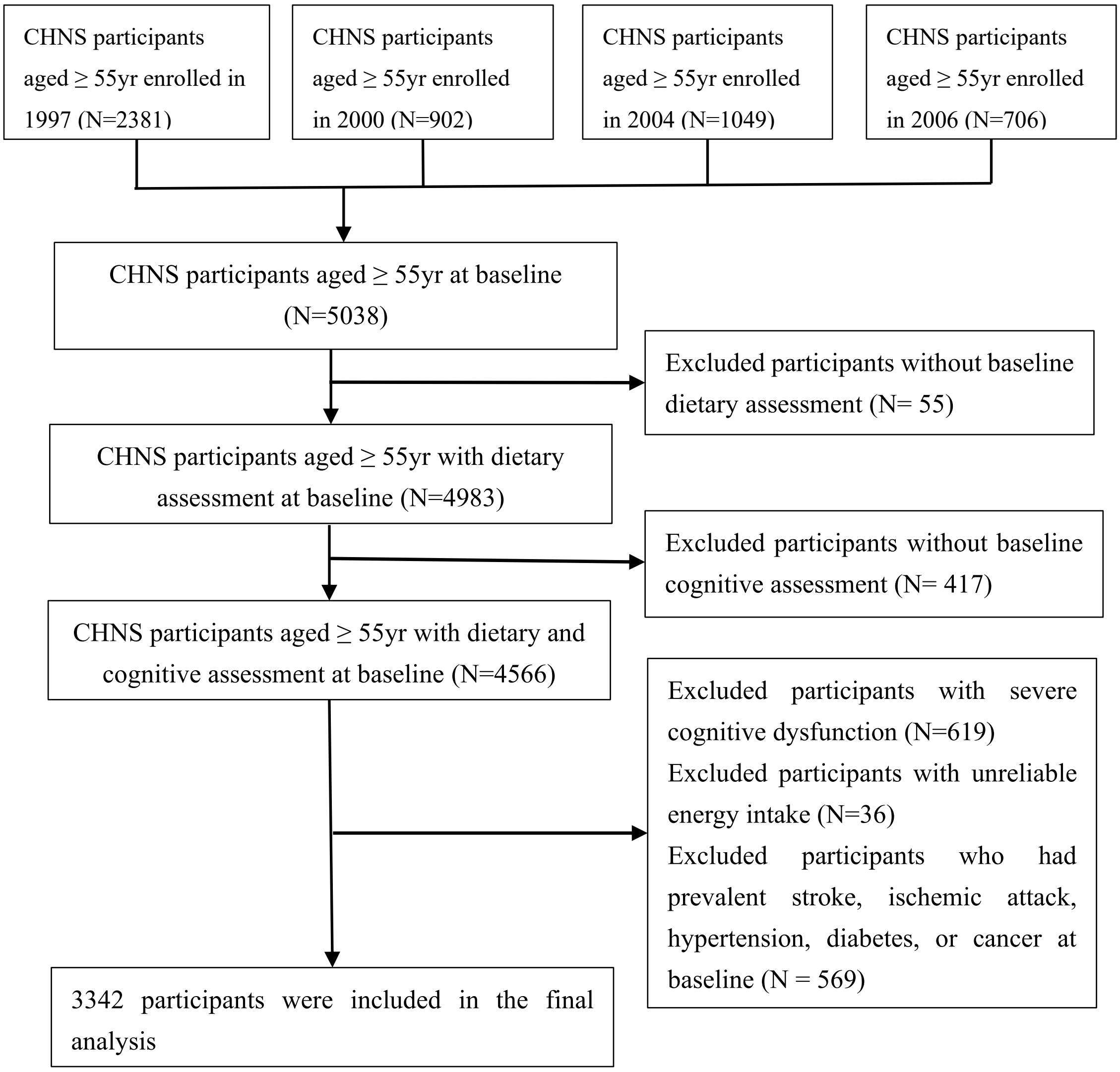

Figure S1. Selection of Study Participants

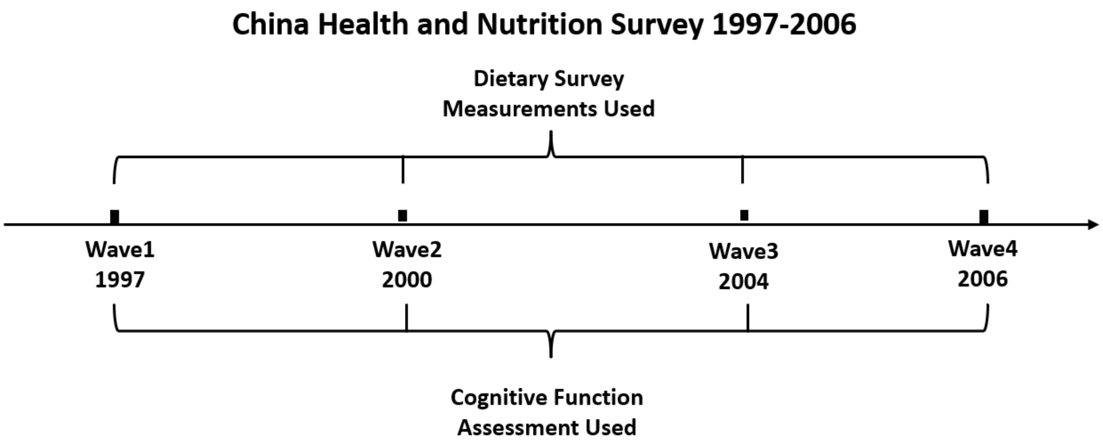

Figure S2. Temporal sequence of the measurements of diet and cognitive function used in the study

Table S1. Cognitive function and its decline associated with the temporal pattern of energy intake (n=3342)

	N	**Difference [95% CI] in cognitive function, points**		**Difference [95% CI] in rate of cognitive decline, points/year**	
		Model 1[a]	Model 2[b]	Model 1[a]	Model 2[b]
Evenly-distributed	1639	Reference	Reference	Reference	Reference
Breakfast-dominant	364	**-1.24 [-1.68, -0.80]**	**-0.94 [-1.37, -0.51]**	0.04 [-0.12, 0.19]	0.03 [-0.12, 0.18]
Lunch-dominant	232	**-1.17 [-1.67, -0.66]**	**-1.18 [-1.67, -0.69]**	-0.12 [-0.31, 0.06]	-0.15 [-0.33, 0.03]
Snack-rich	119	-0.31 [-0.97, 0.34]	**-1.05 [-1.70, -0.40]**	-0.13 [-0.56, 0.31]	-0.15 [-0.57, 0.27]
Dinner-dominant	278	**-1.04 [-1.51, -0.56]**	**-0.97 [-1.43, -0.51]**	-0.06 [-0.21, 0.09]	-0.10 [-0.25, 0.05]
Breakfast-skipping	710	**-1.31 [-1.66, -0.96]**	**-1.32 [-1.66, -0.99]**	**-0.14 [-0.24, -0.04]**	**-0.14 [-0.24, -0.04]**

Mixed-effect linear regression was used to estimate the difference in cognitive function and its decline rate associated with different temporal patterns of energy intake.

[a] adjusted for age and gender;

[b] additionally adjusted for residence (rural or urban), total energy, physical activity (tertiles), smoking status (ever smoked or not), alcohol consumption (currently drinking or not), household income (tertiles), education level (holding high school degree or not), and BMI (<24, 24.0-27.9, ≥28.0 kg/m^2).

Table S2. The cognitive function associated with the temporal pattern of energy intake in the CHNS stratified by gender, age, BMI, residence, and education (n=3342)

	Evenly-distributed	Breakfast-dominant	Lunch-dominant	Snack-rich	Dinner-dominant	Breakfast-skipping	P-interaction
Gender							0.065
Female	Ref	-1.11 [-1.75, -0.48]	-1.39 [-2.12, -0.67]	-0.64 [-1.57, 0.28]	-1.23 [-1.91, -0.55]	-1.46 [-1.96, -0.97]	
Male	Ref	-0.63 [-1.22, -0.03]	-0.75 [-1.42, -0.08]	-1.45 [-2.38, -0.52]	-0.56 [-1.20, 0.07]	-1.04 [-1.51, -0.56]	
Age							0.072
<65 years	Ref	-0.60 [-1.11, -0.09]	-0.72 [-1.29, -0.15]	-0.65 [-1.41, 0.11]	-0.68 [-1.22, -0.14]	-0.99 [-1.39, -0.59]	
≥65 years	Ref	-1.67 [-2.53, -0.82]	-2.00 [-2.99, -1.01]	-2.00 [-3.32, -0.68]	-1.51 [-2.42, -0.59]	-1.95 [-2.60, -1.29]	
BMI							0.029
<24 kg/m^2	Ref	-0.92 [-1.46, -0.39]	-1.15 [-1.75, -0.55]	-0.93 [-1.78, -0.09]	-1.07 [-1.61, -0.53]	-1.50 [-1.90, -1.09]	
≥24 kg/m^2	Ref	-0.92 [-1.67, -0.16]	-0.99 [-1.85, -0.13]	-1.07 [-2.11, -0.03]	-0.52 [-1.42, 0.39]	-0.74 [-1.38, -0.10]	
Residence							0.143
Urban area	Ref	-1.62 [-2.40, -0.84]	-1.30 [-2.09, -0.52]	-1.37 [-2.32, -0.43]	-1.57 [-2.32, -0.82]	-1.49 [-2.01, -0.97]	
Rural area	Ref	-0.61 [-1.13, -0.08]	-0.96 [-1.59, -0.32]	-0.75 [-1.66, 0.16]	-0.56 [-1.15, 0.04]	-1.13 [-1.58, -0.68]	
Education							0.017
≥High School	Ref	-0.24 [-1.44, 0.95]	0.20 [-1.03, 1.42]	-0.90 [-2.04, 0.24]	-0.97 [-2.09, 0.16]	-0.16 [-1.02, 0.71]	
<High School	Ref	-0.99 [-1.46, -0.53]	-1.32 [-1.85, -0.78]	-0.97 [-1.76, -0.18]	-0.88 [-1.39, -0.38]	-1.41 [-1.78, -1.04]	

Table S3. Cognitive decline (points/year) associated with the temporal pattern of energy intake in the CHNS stratified by gender, age, BMI, residence, and education (n=3342)

	Evenly-distributed	Breakfast-dominant	Lunch-dominant	Snack-rich	Dinner-dominant	Breakfast-skipping	P-interaction
Gender							1.000
Female	Ref	-0.06 [-0.29, 0.17]	-0.28 [-0.57, 0.00]	-0.13 [-0.80, 0.55]	-0.22 [-0.44, -0.00]	-0.16 [-0.31, -0.02]	
Male	Ref	0.11 [-0.09, 0.32]	-0.06 [-0.30, 0.18]	-0.19 [-0.74, 0.36]	-0.02 [-0.23, 0.19]	-0.11 [-0.25, 0.03]	
Age							0.014
<65 years	Ref	-0.03 [-0.21, 0.16]	-0.30 [-0.53, -0.06]	-0.18 [-0.68, 0.32]	-0.14 [-0.32, 0.04]	-0.14 [-0.26, -0.01]	
≥65 years	Ref	0.16 [-0.11, 0.42]	-0.02 [-0.32, 0.28]	-0.09 [-0.89, 0.72]	-0.10 [-0.37, 0.18]	-0.17 [-0.35, 0.01]	
BMI							0.686
<24 kg/m^2	Ref	0.04 [-0.14, 0.21]	-0.18 [-0.41, 0.04]	-0.08 [-0.60, 0.43]	-0.12 [-0.29, 0.05]	-0.15 [-0.27, -0.03]	
≥24 kg/m^2	Ref	0.08 [-0.22, 0.38]	-0.15 [-0.46, 0.16]	-0.32 [-1.09, 0.45]	-0.15 [-0.49, 0.19]	-0.09 [-0.28, 0.10]	
Residence							0.996
Urban area	Ref	0.04 [-0.23, 0.31]	-0.16 [-0.45, 0.13]	-0.50 [-1.10, 0.10]	-0.11 [-0.36, 0.14]	-0.18 [-0.34, -0.03]	
Rural area	Ref	0.04 [-0.15, 0.22]	-0.18 [-0.41, 0.05]	0.14 [-0.47, 0.75]	-0.12 [-0.31, 0.07]	-0.13 [-0.26, 0.01]	
Education							0.300
≥High School	Ref	0.36 [-0.10, 0.83]	0.11 [-0.38, 0.61]	-0.43 [-1.30, 0.45]	-0.07 [-0.48, 0.34]	-0.06 [-0.32, 0.20]	
<High School	Ref	0.01 [-0.15, 0.17]	-0.20 [-0.40, -0.01]	-0.11 [-0.59, 0.37]	-0.12 [-0.29, 0.04]	-0.15 [-0.26, -0.04]	

Table S4. Sensitivity analyses for the relations of the temporal pattern of energy intake with cognitive function (CF) and its decline (CD) in CHNS

	Evenly-distributed	Breakfast-dominant	Lunch-dominant	Snack-rich	Dinner-dominant	Breakfast-skipping
Verbal memory domain[a]						
N	1639	364	232	119	278	710
Verbal memory function	Reference	**-0.65 [-0.99, -0.31]**	**-0.88 [-1.27, -0.50]**	**-1.01 [-1.52, -0.50]**	**-0.78 [-1.15, -0.42]**	**-1.02 [-1.28, -0.75]**
Verbal memory decline	Reference	0.08 [-0.04, 0.20]	-0.10 [-0.24, 0.04]	-0.17 [-0.51, 0.17]	-0.04 [-0.16, 0.08]	-0.06 [-0.14, 0.02]
Controlling for cluster effect[b]						
N	1639	364	232	119	278	710
CF	Reference	**-0.93 [-1.36, -0.50]**	**-1.19 [-1.68, -0.70]**	**-0.93 [-1.58, -0.27]**	**-1.03 [-1.49, -0.56]**	**-1.40 [-1.74, -1.06]**
CD	Reference	0.04 [-0.11, 0.19]	-0.17 [-0.35, 0.02]	-0.14 [-0.57, 0.28]	-0.09 [-0.24, 0.06]	**-0.14 [-0.24, -0.04]**
Adjusting CFPS score[c]						
N	1639	364	232	119	278	710
CF	Reference	**-0.91 [-1.34, -0.48]**	**-1.17 [-1.66, -0.68]**	**-1.02 [-1.67, -0.37]**	**-1.00 [-1.46, -0.54]**	**-1.34 [-1.68, -1.00]**
CD	Reference	0.03 [-0.12, 0.18]	-0.16 [-0.35, 0.02]	-0.13 [-0.56, 0.29]	-0.09 [-0.24, 0.06]	**-0.14 [-0.24, -0.04]**
Lagged for one wave[a]						
N	716	229	185	99	199	483
CF	Reference	-0.45 [-1.09, 0.20]	**-0.90 [-1.60, -0.20]**	-0.63 [-1.53, 0.27]	**-0.78 [-1.45, -0.11]**	**-1.43 [-1.94, -0.91]**
CD	Reference	-0.10 [-0.43, 0.24]	0.01 [-0.39, 0.42]	-0.06 [-0.75, 0.62]	-0.17 [-0.53, 0.18]	-0.18 [-0.41, 0.04]
Limited to those who had consistent TPEI[a]						
N	343	55	19	15	33	166
CF	Reference	-0.05 [-1.05, 0.95]	-1.14 [-2.57, 0.29]	0.23 [-1.62, 2.09]	-0.35 [-1.50, 0.80]	**-0.99 [-1.65, -0.32]**
CD	Reference	0.24 [-0.07, 0.56]	-0.16 [-0.63, 0.31]	0.92 [-0.06, 1.91]	-0.19 [-0.52, 0.14]	-0.09 [-0.27, 0.10]

The mixed-effect linear regression model was used to estimate the changes of cognitive function and its decline rate for temporal pattern of energy intake. β (95% CI) was presented.

[a] This model was the full model adjusted for age, gender, residence (rural or urban), total energy, physical activity (tertiles), smoking status (ever smoked or not), alcohol consumption (currently drinking or not), household income (tertiles), education level (below high school or not), and BMI (<24, 24.0-27.9, ≥28.0 kg/m^2).

[b] This model was additionally adjusted for communities in rural or urban areas besides the full model.

[c] This model was additionally adjusted for CFPS score besides the full model.

Table S5. Cognitive Function and its Decline Associated with Priori-Based Temporal Pattern of Energy Intake in the China Health and Nutrition Study (n=3342)

	β (95% CI)	p-value
Cognitive Function, points		
Evenly-distributed (ED, n=512)	Reference	
Breakfast-dominant (BD, n=343)	-0.69 [-1.22, -0.16]	0.011
Lunch-dominant (LD, n=1157)	-0.87 [-1.28, -0.45]	<0.001
Dinner-dominant (DD, n=1330)	-0.66 [-1.08, -0.24]	0.002
Cognitive Decline, points/year		
ED * Year (n=512)	Reference	
BD * Year (n=343)	0.03 [-0.15, 0.20]	0.77
LD * Year (n=1157)	-0.01 [-0.13, 0.10]	0.807
DD * Year (n=1330)	-0.06 [-0.18, 0.06]	0.313

The mixed-effect linear regression model was used to estimate the changes in cognitive function and its decline rate for the temporal pattern of energy intake.

The multivariable model was adjusted for age, gender, residence (rural or urban), total energy, physical activity (tertiles), smoking status (ever smoked or not), alcohol consumption (currently drinking or not), household income (tertiles), education level (below high school or not), and BMI (<24, 24.0-27.9, ≥28.0 kg/m^2).

Table S6. Cognitive function and its decline associated with energy intake from three eating occasions (quartiles) in the China Health and Nutrition Study (n=3342)

	Morning Eating Occasions β (95% CI)	p-value	Afternoon Eating Occasions β (95% CI)	p-value	Evening Eating Occasions β (95% CI)	p-value
Cognitive Function, points						
Quartile 1 (Q1)	Reference		Reference		Reference	
Quartile 2 (Q2)	1.59 [1.22, 1.95]	<0.001	0.59 [0.22, 0.96]	0.002	0.62 [0.25, 0.98]	0.001
Quartile 3 (Q3)	1.74 [1.37, 2.11]	<0.001	0.74 [0.37, 1.11]	<0.001	0.15 [-0.22, 0.51]	0.437
Quartile 4 (Q4)	1.14 [0.77, 1.51]	<0.001	-0.36 [-0.73, 0.01]	0.054	-0.77 [-1.14, -0.41]	<0.001
Cognitive Decline, points/year						
Q1*Year	Reference		Reference		Reference	
Q2*Year	0.14 [0.01, 0.27]	0.035	0.04 [-0.07, 0.15]	0.506	0.07 [-0.03, 0.18]	0.182
Q3*Year	0.15 [0.02, 0.28]	0.024	0.02 [-0.09, 0.13]	0.769	0.02 [-0.09, 0.13]	0.704
Q4*Year	0.13 [-0.01, 0.26]	0.061	-0.15 [-0.27, -0.03]	0.015	-0.04 [-0.16, 0.08]	0.537

The mixed-effect linear regression model was used to estimate the changes of cognitive function and its decline rate for Quintiles 2–4 versus Quintile 1 of energy intakes in the morning, noon anf afternoon, and evening. The multivariable model was adjusted for age, gender, residence (rural or urban), total energy, physical activity (tertiles), smoking status (ever smoked or not), alcohol consumption (currently drinking or not), household income (tertiles), education level (below high school or not), and BMI (<24, 24.0-27.9, ≥28.0 kg/m^2).

Table S7. Cognitive function and its decline associated with snack intake in the China Health and Nutrition Survey (n=3342)

	Cognitive Function β (95% CI)	P value	Cognitive Decline β (95% CI)	P value
Morning Snack	-0.58 [-1.26, 0.10]	0.096	-0.06 [-0.25, 0.14]	0.556
Afternoon Snack	-0.03 [-0.58, 0.52]	0.91	0.17 [0.01, 0.33]	0.037
Evening Snack	0.55 [0.08, 1.02]	0.021	0.18 [0.04, 0.32]	0.012

The mixed-effect linear regression model was used to estimate the association of snack intake with cognitive function. The multivariable model was adjusted for age, gender, residence (rural or urban), total energy, physical activity (tertiles), smoking status (ever smoked or not), alcohol consumption (currently drinking or not), household income (tertiles), education level (below high school or not), and BMI (<24, 24.0-27.9, ≥28.0 kg/m2).

Supplementary References

1. Crimmins EM, Kim JK, Langa KM, Weir DR: **Assessment of cognition using surveys and neuropsychological assessment: the Health and Retirement Study and the Aging, Demographics, and Memory Study.** *J Gerontol B Psychol Sci Soc Sci* 2011, **66 Suppl 1:**i162-171.
2. Shang X, Hill E, Li Y, He M: **Energy and macronutrient intakes at breakfast and cognitive declines in community-dwelling older adults: a 9-year follow-up cohort study.** *Am J Clin Nutr* 2021, **113:**1093-1103.
3. Yang YX, Wang GY, Pan XC: *China food composition tables.* Beijing: Beijing Medical University Press; 2009.
4. Yao M, McCrory MA, Ma G, Tucker KL, Gao S, Fuss P, Roberts SB: **Relative influence of diet and physical activity on body composition in urban Chinese adults.** *Am J Clin Nutr* 2003, **77:**1409-1416.
5. Khanna N, Eicher-Miller HA, Boushey CJ, Gelfand SB, Delp EJ: **Temporal Dietary Patterns Using Kernel k-Means Clustering.** *Ism* 2011, **2011:**375-380.
6. Plassman BL, Welsh KA, Helms M, Brandt J, Page WF, Breitner JC: **Intelligence and education as predictors of cognitive state in late life: a 50-year follow-up.** *Neurology* 1995, **45:**1446-1450.
7. Lam BCP, Haslam C, Steffens NK, Yang J, Haslam SA, Cruwys T, Pachana NA: **Longitudinal Evidence for the Effects of Social Group Engagement on the Cognitive and Mental Health of Chinese Retirees.** *J Gerontol B Psychol Sci Soc Sci* 2020, **75:**2142-2151.
8. Luo L, Wang G, Zhou H, Zhang L, Ma CX, Little JP, Yu Z, Teng H, Yin JY, Wan Z: **Sex-specific longitudinal association between baseline physical activity level and cognitive decline in Chinese over 45 years old: Evidence from the China health and retirement longitudinal study.** *Aging Ment Health* 2021:1-9.
9. Huang Y, Tian X: **Food accessibility, diversity of agricultural production and dietary pattern in rural China.** *Food Policy* 2019, **84:**92-102.
10. Shang X, Flehr A, Fang Y, He M: **Meal patterns and incident hypertension in community-dwelling middle-aged adults: an 11-year follow-up cohort study.** *J Hypertens* 2021, **39:**1393-1401.
11. Pinheiro JB, D.; DebRoy, S.; Sarkar, D.; R Core Team: **nlme: Linear and Nonlinear Mixed Effects Models.** In *R package version 31-144*2020.
